# Beyond priming: a sequential, feedback-guided adjuvant framework for therapeutic cancer peptide vaccines in immunologically cold tumors

**DOI:** 10.3389/fimmu.2026.1835816

**Published:** 2026-05-19

**Authors:** Corey K. Goldman

**Affiliations:** 1 Department of Cardiovascular Medicine, MedAlliance, Bronx, NY, United States; 2 Leon H. Charney Division of Cardiology, Department of Medicine, NYU Grossman School of Medicine, NYU Langone Health, New York, NY, United States

**Keywords:** Adjuvant design, cancer vaccines, CXCR3, IL-15, IL-21, IL-7, immunologically cold tumors, peptide vaccines

## Abstract

Therapeutic cancer vaccines can generate measurable antigen-specific immune responses in humans, yet tumor regression is often incomplete, inconsistent, or short-lived. In immunologically cold tumors, this pattern may reflect not an absolute inability to prime immunity, but difficulty advancing induced immunity through the full sequence required for tumor control. Peripheral blood responses may be real and still be biologically inadequate if they contract early, fail to acquire productive trafficking programs sufficient for tissue entry, lose functional competence under chronic antigen stress, or remain constrained by the suppressive tumor microenvironment. The manuscript advances a sequential, feedback-guided adjuvant framework in which peptide vaccination remains the backbone but is preceded and followed by distinct support phases. A Phase 0 immune-readiness step, potentially using IL-7 (e.g., CYT107), is intended to improve the baseline substrate before antigen exposure. Phase 1 priming uses peptide vaccination on a commonly used adjuvant backbone such as Montanide ISA-51 or poly-ICLC (Hiltonol), while radiation and/or STING-based strategies are treated as context-dependent enhancers rather than replacements for priming. IL-15-centered consolidation is then used to support expansion and persistence. A formal trafficking assessment follows so that blood-only success is not overinterpreted. IL-21 is positioned later as a persistence- and quality-support cytokine when response quality declines. The framework also addresses why otherwise rational protocols can fail at the chemokine-trafficking step: CXCR3-dependent tumor entry is not interchangeable with generic inflammation, CCR5 biology is context dependent, and IL-12, although biologically attractive and previously tested as a vaccine adjuvant, is best viewed here as an optional, context-specific amplifier rather than a universal backbone. Although organized as sequential phases, the framework is intended as a bottleneck-guided and iterative design logic in which phases may overlap, repeat, or be entered in partial parallel depending on the dominant biologic constraint. The central hypothesis is that vaccine programs that progress beyond priming into trafficking-competent and functionally sustained states are predicted to correlate more closely with disease control than programs judged mainly by early blood immunogenicity.

## 1 Introduction

Therapeutic cancer vaccines rest on a straightforward premise: identify relevant antigens, present them effectively, induce tumor-specific immunity, and convert that immunity into clinically meaningful tumor control. Across peptide vaccines, dendritic-cell vaccines, tumor-cell vaccines, vector-based approaches, and individualized neoantigen platforms, many studies have shown that vaccination can generate measurable immune activity in humans (1–5). Yet the field has also been marked by a persistent pattern: immunogenicity is often demonstrable while regression of established tumors remains delayed, partial, or absent (1–6).

That history should not be reduced to simple failure. Prior vaccine studies established safety, showed that tumors can in principle be rendered immunologically visible, and generated important tools for antigen selection, immune monitoring, and rational combination design (1–5). At the same time, they exposed a deeper problem. In many settings, a detectable immune response has sometimes been treated as though it were itself a sufficient endpoint, even though the biological sequence required for tumor control is substantially longer. Across multiple solid-tumor settings, evidence of immunogenicity or biological activity has not reliably translated into durable clinical benefit (1–6).

Therapeutic cancer vaccination is best understood as a multistep systems problem. Priming is necessary, but priming alone is not enough. A useful response must also persist, traffic, enter tumor tissue, retain functional quality, withstand or escape local suppression, and remain renewable over time. The goal of the present framework is therefore not to replace priming, but to place priming inside a rational staged immunotherapy design sequence that may be especially relevant in immunologically cold tumors and that uses peptide vaccination as its clinical backbone (6).

## 2 Immune response does not equal tumor control

One of the most consequential interpretive errors in prior vaccine work has been overreliance on blood-based readouts. Enzyme-linked immunospot (ELISpot) positivity, intracellular cytokine staining (ICS) for cytokine production, peptide–major histocompatibility complex (pMHC) tetramer detection of antigen-specific T-cell receptors, delayed-type hypersensitivity as an *in vivo* marker of cellular memory, or transient proliferative signals can all indicate that a biological event occurred, but they do not by themselves demonstrate tumor-effective immunity. Classic tissue-immunology studies showed that the type, density, and location of immune cells within tumors often carry more prognostic meaning than systemic measurements alone (7, 8).

This distinction is not semantic. A circulating vaccine-induced population may still fail therapeutically because it contracts rapidly, lacks the right differentiation state, does not acquire a productive homing phenotype, cannot traverse the tumor vascular checkpoint, or becomes dysfunctional after entering tissue. Modified peptide-vaccine studies have further shown that measurable responses may still be only weakly tumor cross-reactive, again helping explain why immunogenicity and tumor regression can diverge (9). The repeated pattern of blood-detectable immunity without commensurate tumor control is therefore not mysterious; it is what one would expect when a multistep biological process is judged mainly at its earliest rung (7–11).

## 3 Why otherwise rational vaccine protocols still fail after priming

The central revision proposed here is that many cancer-vaccine protocols have not failed because priming is useless, but because the induced response commonly stalls after priming. The response may be real, yet too small, too brief, too blood-confined, inadequately trafficked, too dysfunctional, or too inhibited to matter clinically. This shifts the key design question from ‘Did immunity increase?’ to ‘Where in the sequence did the induced response stop progressing?’.

A major bottleneck is trafficking and local engagement. Vaccine-primed cells must do more than circulate; they must acquire migratory programs that permit tissue entry and function at relevant tumor sites. The C-X-C motif chemokine receptor 3 (CXCR3)/CXCL9-CXCL11 axis is central to T helper 1-polarized CD4-cell and effector CD8-cell trafficking, tissue extravasation, and local immune amplification (12–14). In a tumor vascular-checkpoint model, CXCR3 signaling was nonredundant for productive tumor entry and therapeutic efficacy, whereas CCR5 was not sufficient to substitute for CXCR3 in CD8 effectors (12). This helps explain why prior protocols can look persuasive in blood yet disappoint in tumors: they generated cells, but not enough cells with the right homing biology in the right chemokine context (11–14).

Trafficking, however, is not chemokine-deterministic. Tumor entry and productive function also depend on vascular activation, adhesion biology, stromal architecture, metabolic constraints, and local suppressive circuits. In this framework, chemokine receptors are treated as important determinants to measure and use in challenging blood-only interpretations rather than as sufficient predictors of tumor engagement.

CC chemokine receptor 5 (CCR5) remains relevant, but its role is context dependent rather than universal. IL-12 can induce CCR5 on activated CD4 and CD8 T cells (15), and CCR5 can contribute to effector recruitment in some settings (16). At the same time, CCR5 biology can intersect with suppressive circuits, including tumor-associated regulatory T-cell accumulation, so simple upregulation of inflammatory chemokines is not automatically favorable (16). For that reason, the present framework treats chemokine biology as something to measure rather than assume: CXCR3-centered trafficking readiness should be assessed explicitly, CCR5 should be interpreted in context, and neither receptor should be presumed to solve the trafficking problem by presence alone.

IL-12 is therefore repositioned here as an optional, context-specific amplifier rather than a mandatory backbone. It should be considered only when monitoring data identify a specific deficit: low CXCR3 expression on circulating effectors, absent or weak Th1 polarization, or imaging and circulating tumor DNA (ctDNA) evidence of poor intratumoral engagement despite adequate priming. When those signals are present, and when delivery can be directed locally or intratumorally to limit systemic toxicity, IL-12 may provide meaningful amplification. When those signals are absent, it should not be added by default (15, 17–19).

## 4 A sequential, feedback-guided adjuvant framework built on peptide-vaccine priming

**Figure 1:**
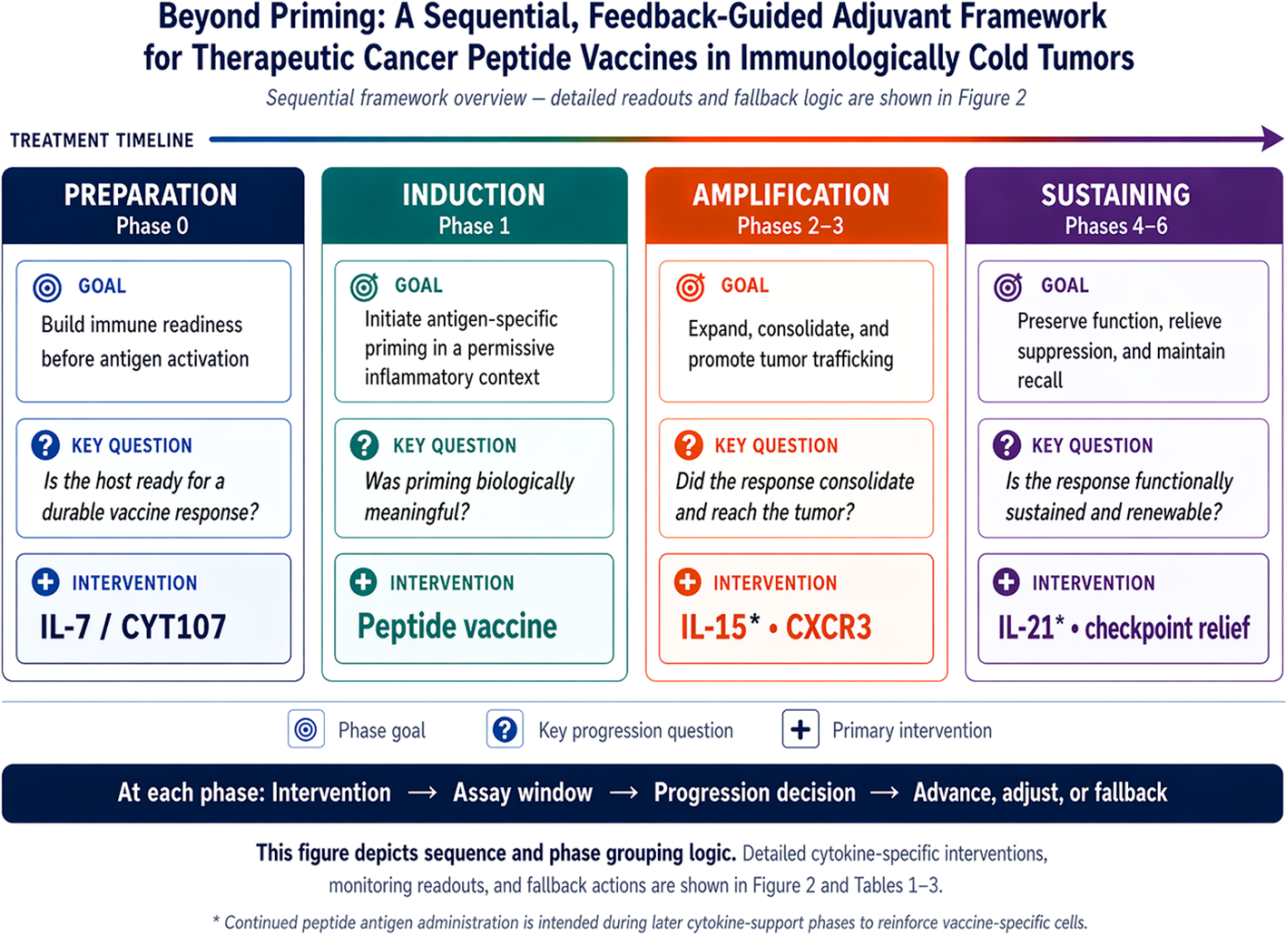
Grouped sequence logic of the staged, feedback-guided adjuvant framework. The figure summarizes four higher-order biological goals - preparation, induction, amplification, and sustaining - while preserving the manuscript’s seven-phase logic conceptually. It emphasizes overall sequence and progression structure rather than detailed phase-specific interventions, monitoring readouts, or fallback actions. Although peptide vaccination is shown explicitly at priming, the proposed framework envisions continued peptide antigen administration during later cytokine-support phases, particularly IL-15 consolidation and IL-21 quality/persistence support, in order to focus these phases on vaccine-specific cells.

The framework proposed here is intentionally staged. It does not assume that every patient requires every element in identical fashion, nor that cytokines operate in a perfectly linear series. It is a disciplined translational architecture that matches specific interventions to predictable bottlenecks in the evolution of an antitumor response. **Figure 1** summarizes the sequence logic, and **Table 1** provides the phase-by-phase scaffold used throughout the article.

**Table 1:** Summary of the proposed sequential framework.

Stage	Primary goal	Lead interventions	Key question
Phase 0	Raise immune readiness before priming	IL-7/CYT107-style conditioning	Is the host prepared to mount a durable vaccine response?
Phase 1	Initiate antigen-specific recognition with adequate inflammatory context	Peptide vaccine with a commonly used adjuvant backbone such as Montanide ISA-51 or poly-ICLC (Hiltonol); radiation and/or STING as context-dependent enhancers	Was priming biologically meaningful rather than merely detectable?
Phase 2	Expand and consolidate the response	IL-15-centered support with continued peptide antigen administration	Did the post-priming response avoid early contraction and begin to consolidate?
Phase 3	Improve tumor access and local engagement	Trafficking-readiness assessment; CXCR3-centered homing logic; selective CCR5 interpretation; context-specific IL-12 or local inflammatory amplification when biologically justified; tissue surrogates when feasible	Can the response get to the tumor and show evidence of local relevance?
Phase 4	Preserve competence and useful response quality	IL-21-centered focusing with continued peptide antigen administration	Is the response qualitatively useful under continued antigen stress?
Phase 5	Release inhibitory brakes	Checkpoint and/or suppressive-axis interventions	Is the induced response still being neutralized by the microenvironment?
Phase 6	Sustain durable control	Boosters, delayed support, maintenance monitoring	Is the response renewable and durable?

As a Hypothesis and Theory contribution, the sequence is presented as a coherent, testable design logic rather than as a fixed regimen. Individual elements (i.e., IL-7/CYT107 conditioning, peptide priming, IL-15-centered consolidation, IL-21-centered quality support, and checkpoint blockade) have supporting evidence in separate contexts, but the full integrated sequence remains to be evaluated prospectively.

An illustrative working sequence is: Phase 0 immune preparation before priming; Phase 1 antigen priming with a peptide-vaccine backbone; Phase 2 expansion and consolidation; Phase 3 trafficking and local engagement; Phase 4 functional focusing and protection from decline; Phase 5 suppression relief; and Phase 6 maintenance. The exact contents of each phase can vary, but the order is intentional because the biological requirements for durable tumor control also tend to emerge in order.

The sequential architecture described here is intended as a decision-guided scaffold rather than a rigid one-way protocol. Progression through the framework should be determined by the dominant biological bottleneck identified during treatment, and phases may overlap, begin in partial parallel, or require iterative re-entry. In practice, some trafficking-linked features may begin to emerge during priming itself, particularly when the priming context is strongly T helper 1-polarized, even if their adequacy for productive tumor engagement is assessed later in the framework. For example, detection of vaccine-induced antigen-specific T cells without adequate numerical or phenotypic consolidation would support renewed Phase 2 cytokine augmentation rather than simple forward progression. Conversely, expansion of circulating effector populations without acquisition of a trafficking-ready phenotype or without favorable ctDNA or imaging trends would suggest a Phase 3 bottleneck, prompting emphasis on tumor engagement rather than additional expansion alone. In selected patients with evidence of pre-existing T-cell inflammation or checkpoint-ligand expression, suppression-relief strategies may also be introduced earlier rather than reserved uniformly as a terminal intervention. In this sense, sequence refers to the logic of bottleneck resolution, not to an invariant calendar schedule.

The framework also generates explicit testable predictions. Programs that progress beyond priming into later phases - particularly trafficking-linked phenotypes with concordant ctDNA stabilization or decline - are predicted to correlate more closely with disease control than programs that generate blood-detectable priming without evidence of later-phase progression. Likewise, selectively adding later-phase support to a declining but already primed response would be expected to be more efficacious than escalating early phase activators in a response that never consolidated in the first place.

## 5 Pre-treatment stratification and tumor-context assignment

Before immune preparation begins, the dominant tumor-context bottleneck should be assigned rather than treating all immunologically cold tumors as biologically equivalent. Some tumors are true immune deserts with little endogenous infiltration or inflammatory tone; some are stromally excluded; some are vascularly excluded despite inducible immunity; and others are partially inflamed but remain functionally constrained by suppressive signaling. These states are expected to differ in the biological step most likely to limit vaccine efficacy and therefore in how strongly each phase of the framework should be emphasized. Desert tumors are the most likely to require the full preparatory and priming sequence. Excluded tumors may still require intact priming and consolidation, but with earlier emphasis on trafficking, vascular access, stromal barriers, or local inflammatory support around Phase 3. By contrast, partially inflamed tumors with evidence of pre-existing T-cell engagement or checkpoint-ligand expression may justify earlier integration of suppression-relief strategies rather than treating checkpoint blockade as uniformly late in every case (20, 21).

**Figure 2:**
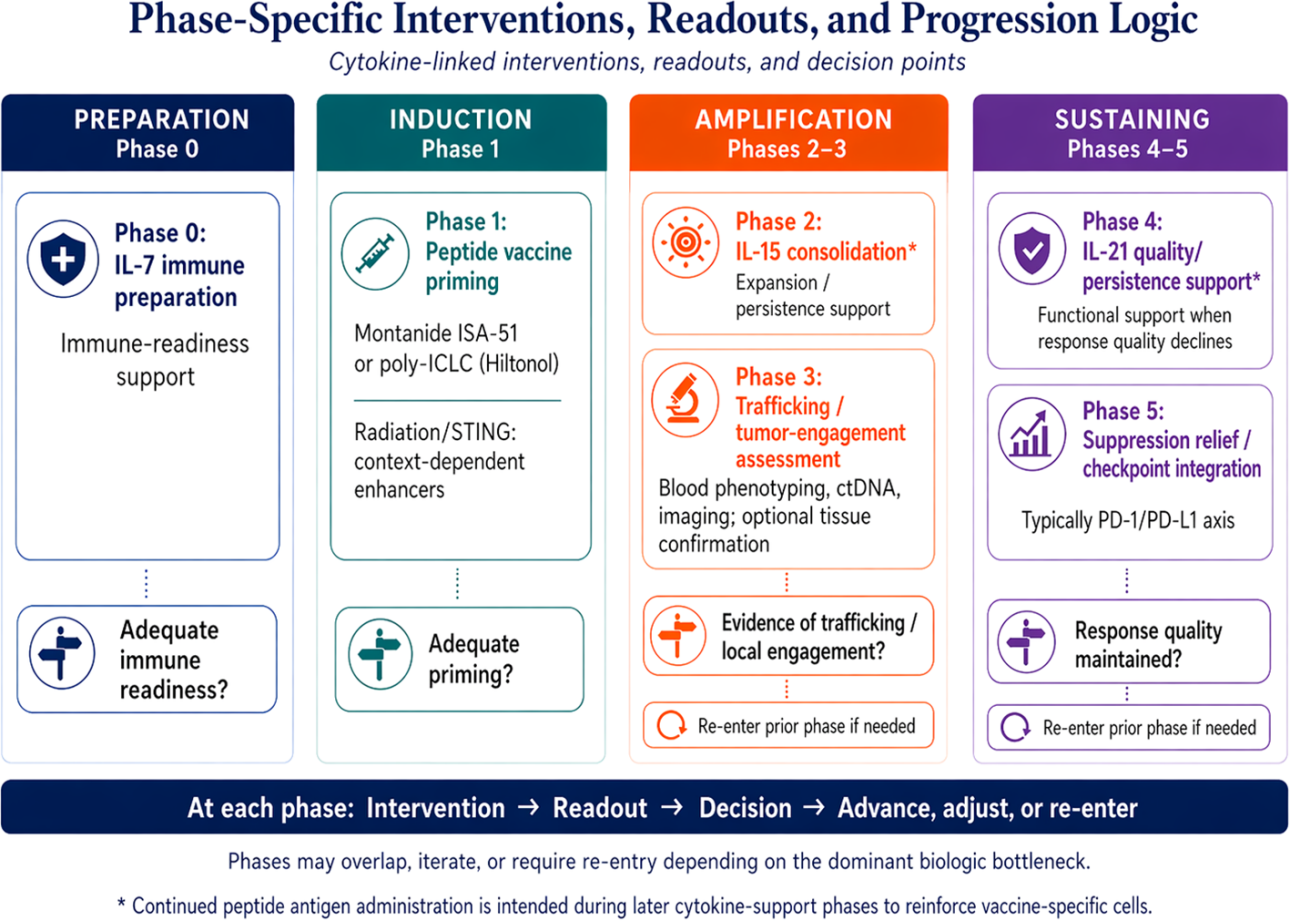
Pre-treatment stratification layer for phase prioritization in immunologically cold tumors. Before entry into the staged cancer vaccine framework, tumors undergo tumor-context assignment and a parallel antigen-fitness review. Practical tumor-context categories include immune desert, stromal exclusion, vascular exclusion, and partially inflamed but suppressed states. These categories help identify the dominant bottleneck and determine which phases of the staged framework should receive greatest emphasis. In parallel, antigen fitness should be reviewed for expression adequacy, heterogeneity, clonality, HLA suitability, and escape/loss risk. The figure is intended as a decision-oriented stratification aid rather than a fixed algorithm; subsequent phases may still overlap, iterate, or require re-entry depending on monitoring results. HLA, human leukocyte antigen.

Where available, this assignment can be strengthened by transcriptomic, multi-omic, or spatial data, including T-cell-inflamed or exclusion signatures, antigen-presentation competence, and vascular or stromal features. Tumor mutational burden and related genomic features may also contribute, but no single biomarker should be treated as a universal gatekeeper across tumor types. A parallel antigen-fitness review is equally important. No sequencing framework can overcome weak antigen expression, marked heterogeneity, subclonality, HLA mismatch, or early escape risk. Vaccination against subclonal neoantigens carries the specific risk of immune-mediated selection pressure that accelerates outgrowth of escape variants; antigen selection should therefore prioritize clonal targets present across the dominant tumor population where feasible (22). Antigen selection and adjuvant sequencing should therefore be treated as equally important, but distinct design problems: one defines what the response is directed against, and the other defines how far that response is advanced toward tumor control. **Figure 2** provides a compact decision-oriented stratification aid for this pre-treatment layer (20–23).

### 5.1 Phase 0: immune preparation before priming

A neglected but clinically important question in cancer vaccination is whether the host is adequately prepared before priming begins. Patients with advanced cancer may have lymphopenia, impaired T-cell repertoire breadth, treatment-related depletion, or a dysfunctional homeostatic environment. Trying to vaccinate under those conditions may be biologically possible, yet still suboptimal for generating durable, high-quality responses.

This is the rationale for a Phase 0 lead-in. The aim is not to replace antigen specificity, but to improve the baseline state in which priming will occur. IL-7 is especially attractive here because, among common gamma-chain cytokines, it is better aligned with repertoire support, survival signaling, and less differentiated T-cell maintenance than with terminal effector overdrive (24). Human feasibility support for this concept is strengthened by CYT107-based studies, which showed peripheral CD4 and CD8 expansion and suggested a more favorable early immune substrate around subsequent vaccine or checkpoint-based interventions (25, 26). In this framework, IL-7 or CYT107 is therefore positioned as preparation rather than activation: a means of improving the substrate before antigen-specific priming begins.

### 5.2 Phase 1: priming on a peptide-vaccine backbone with context-dependent coincident activation

Antigen priming remains central in this model. The working formulation uses peptide vaccination with a commonly used adjuvant backbone such as Montanide ISA-51 or poly-ICLC (Hiltonol) so that antigen-specific recognition can be induced and amplified in a controlled fashion. Although platform-specific variation is possible, the core principle is constant: without priming, there is no vaccine-generated population to expand, focus, or support later.

In this framework, peptide vaccination is not restricted to Phase 1 priming alone. After initial priming, subsequent cytokine-support phases are also intended to occur with continued peptide antigen administration, ideally in local/coordinated co-administration, so that expansion and functional support remain preferentially focused on vaccine-specific cells rather than broadly activated bystander populations.

Radiation and/or stimulator of interferon genes (STING)-based strategies remain in the model, but as context-dependent enhancers rather than replacements for the priming decision. Their intended role is to improve dendritic-cell activation, type I interferon tone, and the quality of the priming context (10, 27–30). If they improve priming quality without unacceptable toxicity, they can help; if they do not, they should not be carried forward simply by tradition. In other words, coincident activators are supportive tools, not the core of the framework.

### 5.3 Phase 2: expansion and consolidation

Once priming has occurred, the response must be expanded and protected from premature contraction. IL-15 is the most coherent lead candidate in the present framework because it supports activated NK-cell and CD8-memory compartments without the repertoire-broadening emphasis associated with IL-7 (24). Early human IL-15 and IL-15 receptor-agonist studies provide empirical support for this positioning, showing marked activation or expansion of NK and CD8-associated compartments and establishing combination feasibility in advanced cancer settings (31–34).

The purpose of this phase is not merely to increase counts. It is to consolidate a newly generated response into a therapeutically meaningful compartment that retains proliferative reserve and memory potential. A priming program that generates expandable yet immediately short-lived cells may be immunogenic without being clinically useful. Phase 2 therefore asks whether the response has become sufficiently numerous and durable to matter, not simply whether it appeared.

### 5.4 Phase 3: trafficking and local engagement

This stage is one of the article’s central additions. Blood-based success should not be equated with tumor-level success unless there is at least some biological evidence that induced cells can acquire and express the trafficking programs needed for tissue entry. The core human-study readouts here should remain clinically realistic: blood-based phenotyping for CXCR3, CCR5, CD49d, and lymphocyte function-associated antigen-1 (LFA-1), used as peripheral surrogates of adhesion and vascular-engagement capacity; ctDNA trend; and imaging, with tissue confirmation reserved for optional translational cohorts rather than routine serial protocol biopsies (7, 8, 12–14). A useful Phase 3 precedent comes from intratumoral plasmid IL-12 electroporation, which in triple-negative breast cancer increased a *CXCR3*-associated gene signature, upregulated trafficking-related chemokine biology, and in patients linked post-treatment enrichment of that signature to increased CD8+ tumor infiltration (35).

The conceptual point is simple but important: a response that cannot traverse the trafficking step is biologically unfinished. For that reason, trafficking cannot be treated as an afterthought or a purely correlative issue. To the extent that blood-based support and anatomic readout can be integrated, they should be used prospectively to test whether expansion, inflammatory amplification, and selected focusing signals actually produce tumor-capable immunity rather than a blood-only immune signal.

### 5.5 Phase 4: functional focusing and protection from decline

Even after expansion and apparent homing readiness, the response may still fail because induced cells lack the qualitative features needed for repeated productive tumor engagement. Chronic antigen exposure can drive contraction, accelerating functional decline, or susceptibility to exhaustion. Quantity without quality is one of the quieter reasons vaccine programs stall.

IL-21 is positioned here as a late, quality-preserving and persistence-support cytokine rather than an indiscriminate booster. Early human studies in melanoma and renal cell carcinoma showed that recombinant IL-21 is biologically active *in vivo*, with NK- and CD8-cell activation and signals of clinical activity in selected settings (36–38). At the same time, IL-21 biology is context dependent rather than uniformly favorable across all differentiation states (39). In the present model, IL-21 is therefore reserved for a late phase in which a response already exists but its qualitative features are deteriorating; it is not proposed as a default early cytokine or a universal solution.

### 5.6 Phase 5: suppression relief

No vaccine framework is complete if it ignores the suppressive tumor microenvironment. A response that has been prepared, primed, expanded, and made tissue-capable may still fail if inhibitory signaling remains dominant. This is where checkpoint blockade and other suppressive-axis interventions enter the sequence. Their role is not to substitute for poor priming, but to release a response that already has meaningful substrate.

This sequencing matters especially in cold tumors. Checkpoint blockade alone may underperform when there are too few antigen-specific effector cells to rescue. The same intervention can look very different when layered onto a response that has already progressed through readiness, priming, expansion, and tissue-capability steps (6). The present framework therefore treats suppression relief as a downstream release phase rather than the default first move.

### 5.7 Phase 6: maintenance

Durable control requires durability of the response itself. A short burst of immune activity is unlikely to suffice if contraction follows before durable tumor control is established. Maintenance may include booster vaccination, delayed cytokine support, continued checkpoint therapy in selected cases, or structured observation if a renewable memory state has already been achieved.

This phase is deliberately flexible because the exact maintenance package will vary by tumor type, disease burden, and earlier phase performance. What should not vary is recognition that maintenance is a biological requirement rather than an afterthought. The endpoint is not merely immune motion; it is renewable, recallable, anatomically capable immunity over time.

## 6 Monitoring checkpoints: the intervention–assay–decision cadence

To keep early-phase implementation realistic, the checkpoints and numerical examples below are illustrative rather than prescriptive. The assays, numerical examples, and decision points described below are not proposed as therapeutic recommendations or clinical decision rules, but as falsifiable, hypothesis-generating anchors intended to support interpretability in early translational testing of the framework. A minimal viable interpretation set can focus on three questions: (i) was priming biologically meaningful beyond expected within-patient fluctuation, (ii) did the post-priming response consolidate rather than immediately contract, and (iii) is there convergent evidence - direct or surrogate - that the response is becoming tumor-capable rather than remaining blood-confined.

**Figure 3:**
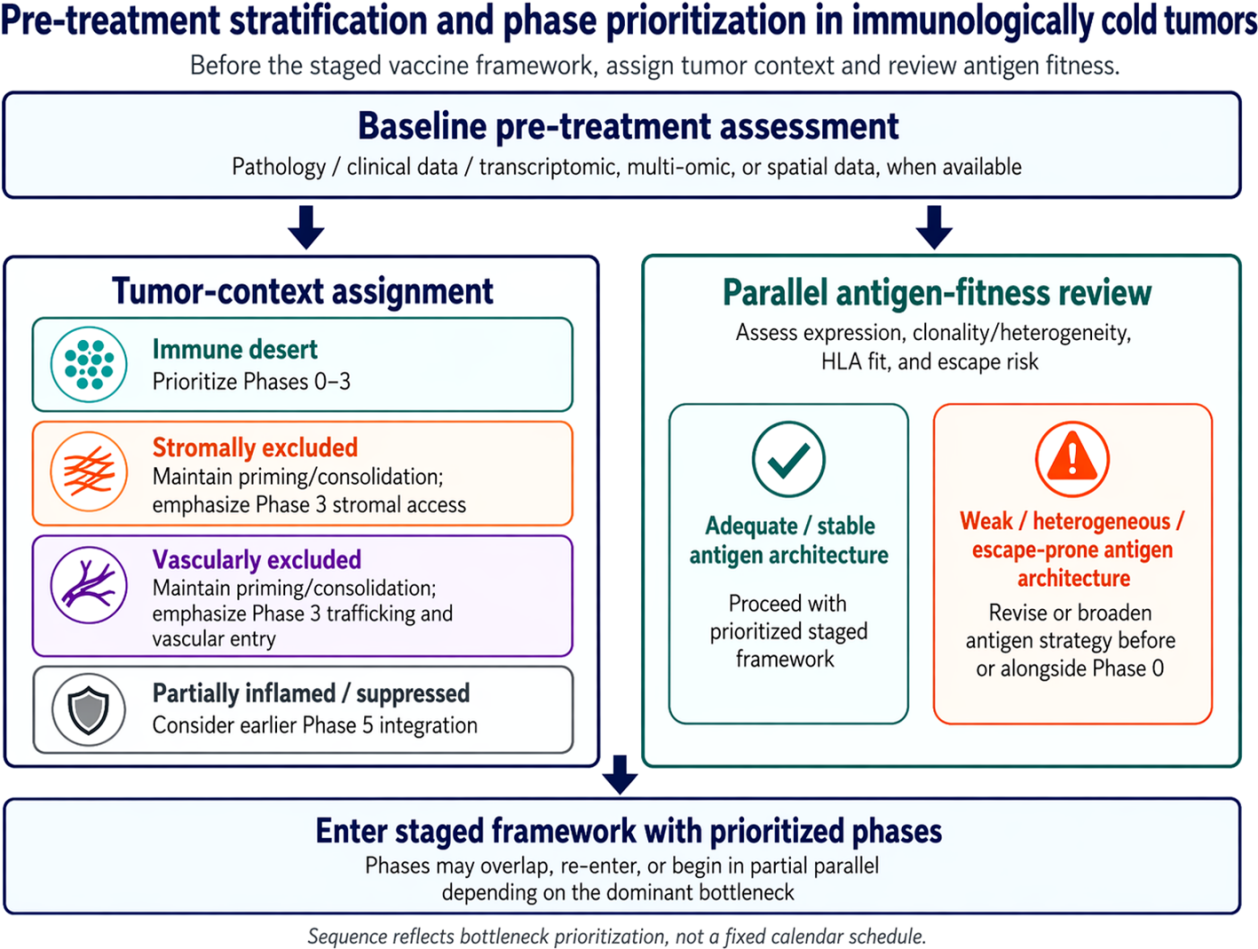
Phase-specific interventions, readouts, and progression logic within the staged framework. The figure organizes the framework into preparation (Phase 0), induction (Phase 1), amplification (Phases 2-3), and sustaining (Phases 4-5), and links each phase to representative interventions, monitoring readouts, and simple progression decisions. For visual simplicity, this workflow figure focuses on Phases 0–5 and principal re-entry logic; Phase 6 maintenance is described in the main text and tables. Asterisked later cytokine-support phases denote continued peptide antigen administration intended to reinforce vaccine-specific cells. poly-ICLC, polyinosinic-polycytidylic acid stabilized with poly-L-lysine and carboxymethylcellulose; STING, stimulator of interferon genes; ctDNA, circulating tumor DNA; PD-1, programmed cell death protein 1; PD-L1, programmed death-ligand 1.

A central feature of this framework is that monitoring is staged. The relevant question is not only whether immunity increased, but whether the response progressed through the biological steps most relevant to therapeutic success: readiness, priming, consolidation, trafficking, functional focusing, suppression relief, and maintenance. **Table 2** summarizes proposed readouts and progression criteria, and **Figure 3** distills the same logic into a compact clinical workflow.

**Table 2:** Proposed operational checkpoints with illustrative numerical and CV-informed heuristics.

Stage	Illustrative readouts	Proposed checkpoint	Why it matters	If checkpoint is not met
Readiness	ALC; CD3/CD4/CD8 counts; naive/TCM/TSCM distribution	≥20% rise or >2x baseline CV with preservation of naive/central-memory features	Avoids priming into a depleted or distorted host	Extend or revise Phase 0; delay priming
Priming	Peptide-specific ELISpot; pMHC tetramer/multimer; ICS for IFN-γ/TNF-α/IL-2	≥2-fold antigen-specific rise and >2x baseline CV	Confirms priming was biologically meaningful	Revise peptide set, formulation, or priming context
Expansion/consolidation	Antigen-specific frequency; Ki-67; CD127/KLRG1; memory-shift metrics	≥30% rise or >2x CV without an early contraction signature	Distinguishes induction from durable consolidation	Prolong or adjust IL-15-centered support
Trafficking/local	CXCR3; selective CCR5; CD49d; LFA-1; chemokine context; ctDNA or imaging concordance	Trafficking-readiness signature plus a concordant downstream surrogate	Prevents blood-only overinterpretation	Add or adjust context-specific IL-12, local inflammatory amplification, or homing support
Functional focusing	Polyfunctional ICS; granzyme B/perforin; exhaustion balance; memory composition	Quality improves beyond threshold and >2x CV without dominant dysfunctional phenotype	Quantity without quality may fail	Introduce or modify IL-21-centered focusing and reassess
Suppression relief	CD8:Treg or effector:suppressor ratio; PD-1/TIM-3/LAG-3 trend; myeloid markers	Directional evidence that inhibitory pressure is falling	Tests whether the response has been released	Layer checkpoint or other suppressive-axis therapy
Maintenance	Serial ELISpot/tetramer persistence; memory retention; ctDNA stability	Retention of ≥50% of peak signal or equivalent durable evidence	Durability is the goal	Revise booster/maintenance strategy

ALC, absolute lymphocyte count; TCM, central memory T cells; TSCM, stem cell memory T cells; ELISpot, enzyme-linked immunospot; pMHC, peptide-major histocompatibility complex; ICS, intracellular cytokine staining; IFN-gamma, interferon-gamma; TNF-alpha, tumor necrosis factor-alpha; CXCR3, C-X-C motif chemokine receptor 3; CCR5, C-C motif chemokine receptor 5; LFA-1, lymphocyte function-associated antigen-1; KLRG1, killer cell lectin-like receptor G1; ctDNA, circulating tumor DNA; Treg, regulatory T cells; PD-1, programmed cell death protein 1; TIM-3, T-cell immunoglobulin and mucin-domain containing-3; LAG-3, lymphocyte activation gene 3; CV, coefficient of variation. Thresholds are included solely as illustrative operational heuristics to enable prospective hypothesis testing and should not be interpreted as validated efficacy criteria.

Practical implementation of these readouts in early-phase studies requires explicit acknowledgment of feasibility and assay limitations. Serial blood-based immune phenotyping is attractive because it is clinically practical and repeatable, but trafficking-related markers such as CXCR3 should be interpreted only under standardized collection, processing, and staining conditions. They are best used as relative longitudinal measures within patients rather than as absolute cross-sectional predictors, and absence of a strong peripheral signal does not exclude intratumoral immune activity. ctDNA trends should likewise be interpreted as complementary disease-context signals rather than as direct surrogates of immune trafficking, particularly in low-shedding tumors or in patients without trackable plasma disease markers. Imaging remains necessary for clinical assessment, yet radiographic changes may lag behind immune activation and can be difficult to interpret during immunotherapy. Accordingly, early-phase implementation of this framework should emphasize practical serial blood monitoring, selective use of ctDNA where informative, standard imaging assessment, and optional tissue-based correlative studies when feasible rather than requiring all modalities in every patient.

An important but deliberately optional extension of this monitoring logic is a paired-biopsy translational layer in selected patients or dedicated correlative cohorts. The practical framework proposed here remains predominantly non-biopsy, and routine serial tumor biopsy is neither required nor likely feasible for most patients because of invasiveness, cost, accessibility, and patient burden. Nevertheless, when blood immunogenicity, ctDNA kinetics, imaging, and clinical outcome are discordant, limited tissue sampling can provide higher-resolution adjudication of where the response failed to progress. In that setting, a baseline biopsy, one early on-treatment biopsy after priming/consolidation but before major late-phase escalation decisions, and an optional progression biopsy when clinically accessible may help distinguish upstream failure of immune preparation, priming, or early consolidation from failure of trafficking/tumor entry, from successful infiltration accompanied by local suppression or functional decline, and from antigen loss or altered antigen-presentation competence despite immune persistence. At minimum, such biopsies could assess H&E architecture, CD8 density and location in the tumor core, invasive margin, and stromal compartment, PD-L1, and tumor-cell identification markers when relevant. Expanded translational panels could include granzyme B and/or Ki-67, FOXP3, selected myeloid-context markers, HLA class I or related antigen-presentation features, and spatial or multiplex methods when feasible. **Table 3** summarizes how selected blood-tissue patterns can help adjudicate the dominant bottleneck in the staged framework (7, 8, 20, 21, 35, 42).

**Table 3:** Optional paired-biopsy translational adjudication matrix for phase assignment when blood and tissue readouts are discordant.

Blood/tissue pattern	Tissue-level interpretation	Dominant phase implication	Practical next emphasis
Blood negative + biopsy desert	No meaningful systemic induction and no local infiltration	Mainly Phases 0–2	Reassess immune readiness, peptide/priming design, and early consolidation; this is not primarily a trafficking problem
Blood positive + biopsy desert	Systemic induction without tumor entry	Mainly Phase 3	Emphasize trafficking/local-engagement support and exclusion biology
Blood weak + biopsy sparse or margin-restricted	Incomplete expansion plus incomplete tissue entry	Combined Phases 2–3	Strengthen consolidation and trafficking/local-entry support
Blood strong + biopsy sparse, stromal, or margin-trapped	Adequate systemic response but ineffective penetration of tumor parenchyma	Mainly Phase 3	Focus on stromal/vascular exclusion, tissue-access barriers, and local inflammatory context
Biopsy inflamed with intratumoral CD8 infiltration but little regression	Later bottlenecks dominate despite tumor entry	Mainly Phases 4–5	Assess functional quality, exhaustion/suppression balance, and release strategies
Immune persistence with continued progression	Consider antigen escape or altered antigen-presentation competence in addition to immune dysfunction	Parallel antigen problem, may coexist with Phases 4–5	Reassess target expression, HLA class I/antigen presentation, and clonal escape risk

Legend: The matrix is intended as an optional translational adjudication aid rather than a mandatory clinical algorithm. “Biopsy desert” refers to absent or negligible CD8 infiltration in both tumor core and invasive margin, whereas “sparse or margin-restricted” patterns refer to limited infiltration confined largely to stroma or the invasive margin. Abbreviations: H&E, hematoxylin and eosin; HLA, human leukocyte antigen; PD-L1, programmed death-ligand 1.

The numerical examples in **Table 2** are proposed operational heuristics rather than validated biostatistical standards. In practice, a progression checkpoint can be defined as change that exceeds both expected intra-patient fluctuation and a biologically meaningful minimum effect size. One practical example is the greater of a fixed threshold or a change exceeding two times the patient’s baseline coefficient of variation for that metric, ideally confirmed on repeat measurement when feasible. These examples are intended to be prospectively calibrated to assay performance, sampling schedule, and disease context rather than treated as universal cutoffs.

The same logic governs what happens when a checkpoint is not met: a stalled phase points to a specific corrective action rather than an open-ended list of options. If consolidation is insufficient, the IL-15 support phase should be extended or adjusted before advancing. If CXCR3 expression and trafficking markers remain low, that would suggest consideration of IL-12 or a local delivery strategy - not progression to the next phase. If the response exists but quality markers are deteriorating, IL-21 becomes the appropriate intervention. If suppressive signals dominate despite an otherwise intact response, checkpoint blockade may be the indicated next step. Staged monitoring therefore does more than track progress; it assigns each failed checkpoint to a biologically matched response and prevents the common error of escalating treatment without first identifying where the sequence broke down.

## 7 Translational implications for cold tumors

This model is especially relevant for immunologically cold tumors because such tumors often begin with insufficient endogenous T-cell infiltration, weak inflammatory tone, and a high likelihood that checkpoint blockade alone will underperform (6). In that setting, the value of a staged vaccine strategy is not only that it can induce antigen-specific cells, but that it can reveal what is missing when those cells fail to matter clinically.

Cold tumors represent a heterogeneous set of indications rather than a single biology. The framework is therefore presented as a transferable staging-and-feedback model that can be adapted to tumor-specific constraints (e.g., dominant suppressive axes, stromal barriers, vascular state, and antigen landscape) while retaining a common interpretive spine.

A practical early-phase study based on this framework would be biomarker intensive and explicitly sequential. The initial aims should be mechanistic before they are ambitious: first, determine whether immune readiness can be improved; second, determine whether priming becomes more meaningful under a strengthened but controlled inflammatory context; and third, determine whether consolidation, trafficking, focusing, and suppression-relief steps move the response into later biological phases rather than leaving it trapped in blood. **Table 4** translates that logic into a compact stage–readout–checkpoint–action–fallback schema.

**Table 4:** Compact stage–readout–checkpoint–action–fallback schema for an early-phase translational study.

Stage	Primary readout	Checkpoint	Action if criterion met	Fallback if not met
Lead-in	ALC; CD3/CD4/CD8; naive/TCM/TSCM shift	Readiness checkpoint met	Proceed to priming	Extend or revise immune-preparation phase
Priming	Antigen-specific ELISpot; tetramer; ICS	Priming checkpoint met	Maintain base platform and move to consolidation	Revise peptide, formulation, or priming enhancer
Consolidation	Antigen-specific frequency; Ki-67; memory-consolidation metrics	Expansion checkpoint met	Advance to trafficking assessment	Prolong or adjust IL-15-centered support
Trafficking check	CXCR3-centered signature; selective CCR5 interpretation; ctDNA/imaging/tissue surrogate	Trafficking checkpoint met	Advance to focusing or suppression relief as indicated	Add context-specific IL-12, local inflammatory amplification, homing support, or reassess timing
Focusing	Polyfunctionality; granzyme B/perforin; exhaustion-balance metrics	Quality checkpoint met	Continue monitoring and move toward release/maintenance	Modify IL-21-centered support and reassess
Release	Effector:suppressor balance; inhibitory-marker trend	Suppression-relief checkpoint met	Continue maintenance program	Layer checkpoint or suppressive-axis intervention
Maintenance	Serial persistence and ctDNA stability	Durability checkpoint met	Observe or boost selectively	Revise booster/maintenance interval

ALC, absolute lymphocyte count; ELISpot, enzyme-linked immunospot; ICS, intracellular cytokine staining; TCM, central memory T cells; TSCM, stem cell memory T cells; CXCR3, C-X-C motif chemokine receptor 3; CCR5, C-C motif chemokine receptor 5; ctDNA, circulating tumor DNA.

This approach should also improve interpretability. Traditional combination protocols often produce an ambiguous answer when they fail. A sequential, checkpoint-guided strategy can instead identify whether readiness never improved, priming never deepened, trafficking never matured, local engagement remained unsupported, or suppression was never adequately relieved. That level of mechanistic accountability may help distinguish biological failure from poor sequencing or overstacked combinations.

## 8 Discussion

The central claim of this article is not that one cytokine or one adjuvant will solve cancer vaccination. The claim is that therapeutic vaccination becomes more rational when the induced immune response is treated as a staged campaign rather than as a single event. Many prior protocols succeeded at generating immune signals but not at moving those signals through the longer biological sequence required for durable tumor control.

The proposed contribution is a staged, monitoring-linked design logic for therapeutic vaccine trials. Rather than cataloging combinations, the framework organizes interventions according to where the induced response appears to stall and links each stage to a next experimental action. That structure is intended to make early trials more interpretable when they succeed only partially or fail.

A further first-order limitation is antigen quality. No staging or adjuvant sequencing can rescue weak antigen choice, limited tumor cross-reactivity, antigen loss, or substantial intratumoral heterogeneity. The framework therefore assumes that antigen selection and validation (including attention to clonality and escape risk) remain parallel design problems; the staging logic improves interpretability and support of a good antigen set but cannot substitute for it.

Antigen evolution and immune escape also represent dynamic failure modes that may emerge after initially successful priming or consolidation. Persistence of vaccine-specific immune signals in blood despite ctDNA progression or renewed tumor growth would be more consistent with antigen loss, clonal outgrowth, or altered antigen presentation than with simple failure of trafficking or functional maintenance. In this sense, discordance between immune persistence metrics and disease kinetics can itself be informative within the framework, helping distinguish immune insufficiency from antigenic escape (22).

The framework also helps reposition several contentious components. IL-7 or CYT107 is not presented as an antitumor effector in its own right, but as a substrate-conditioning tool. IL-15 is not merely a growth factor, but a consolidation strategy supported by early human IL-15 and IL-15 receptor-agonist studies (31–34). More recent clinical combination experience strengthens that logic from a translational standpoint: IL-15-pathway support has been associated with durable antitumor responses in humans, supporting the concept that this stage can contribute to sustained immune activity rather than only transient peripheral activation (40). Complementing this, the QuICC trial combined vaccine, N-803, and checkpoint/TGF-beta blockade within a single clinical platform (41). Together with earlier N-803 plus nivolumab data in NSCLC (34), this combination experience provides direct clinical precedent for the broader staged combination logic proposed here, even though the optimal sequence remains to be defined. IL-21 is not a generic booster, but a late quality-support and persistence-support signal informed by early human biological and clinical studies, used cautiously because its effects are context dependent (36–39). IL-12 is acknowledged as a biologically strong but operationally complex molecule that may help selected patients under selected circumstances, especially around local inflammatory tone and homing support as positioned in Phase 3, but should not be treated as mandatory for every protocol (15, 17–19).

Several limitations should be stated directly. The framework is biologically rational, but it is not yet clinically proven in the exact order and timing proposed. Evidence from human studies is strongest for the Phase 0 IL-7/CYT107 concept and more indirect for several later sequencing decisions. Chemokine biology is not one-dimensional; pathways such as CXCR3 and CCR5 can support effector recruitment in one context and suppressive recruitment or tumor-associated complexity in another (12–16). Routine serial tissue sampling is unlikely to be realistic in most human vaccine studies, so trafficking or local-engagement assessment will often rely on blood phenotyping, ctDNA kinetics, and imaging, with tissue confirmation reserved for optional translational cohorts.

A limited paired-biopsy translational strategy may nevertheless be particularly informative in selected cohorts. Baseline and early on-treatment biopsies, with optional progression biopsy when clinically accessible, can help distinguish absent induction from failed trafficking, intratumoral entry with local suppression or dysfunction, and antigen loss or altered antigen-presentation competence despite immune persistence. In that sense, biopsy is positioned here not as a universal requirement but as a tissue-level adjudication tool for discordant cases and for mechanism-rich early clinical trials (7, 8, 22, 35, 42).

The framework nevertheless yields concrete near-term predictions. Patients whose responses progress beyond priming into later phases - especially trafficking readiness and preserved functional quality - would be expected to show better disease stabilization than patients with similar early blood immunogenicity but no evidence of later-phase progression. In parallel, selectively adding later-phase support to a declining response would be expected to outperform indiscriminate escalation of all components at once.

These uncertainties define the scope of the present article and are consistent with the Hypothesis and Theory format. Its contribution is a staged immunotherapy design framework for therapeutic cancer vaccines in immunologically cold tumors that uses peptide vaccination as its backbone: prepare the host, prime with intent, consolidate what is induced, verify tumor-capable trafficking, protect response quality, relieve suppression when justified, and maintain durable recall.

## Data Availability

The original contributions presented in the study are included in the article/supplementary material. Further inquiries can be directed to the corresponding author.
